# Facile Construction of Hybrid Hydrogels with High Strength and Biocompatibility for Cranial Bone Regeneration

**DOI:** 10.3390/gels8110745

**Published:** 2022-11-17

**Authors:** Shuai Chang, Jiedong Wang, Nanfang Xu, Shaobo Wang, Hong Cai, Zhongjun Liu, Xing Wang

**Affiliations:** 1 Department of Orthopedics, Peking University Third Hospital, Beijing 100191, China; 2 Beijing Key Laboratory of Spinal Disease Research, Peking University Third Hospital, Beijing 100191, China; 3 Engineering Research Center of Bone and Joint Precision Medicine, Ministry of Education, Peking University Third Hospital, Beijing 100191, China; 4 Beijing National Laboratory for Molecular Sciences, Institute of Chemistry, Chinese Academy of Sciences, Beijing 100190, China; 5 University of Chinese Academy of Sciences, Beijing 100049, China

**Keywords:** bone tissue engineering, bioactive glass, hybrid hydrogel, high mechanics, injectability

## Abstract

The significant efforts being made towards the utilization of artificial soft materials holds considerable promise for developing tissue engineering scaffolds for bone-related diseases in clinics. However, most of these biomaterials cannot simultaneously satisfy the multiple requirements of high mechanics, good compatibility, and biological osteogenesis. In this study, an osteogenic hybrid hydrogel between the amine-functionalized bioactive glass (ABG) and 4-armed poly(ethylene glycol) succinimidyl glutarate-gelatin network (SGgel) is introduced to flexibly adhere onto the defective tissue and to subsequently guide bone regeneration. Relying on the rapid ammonolysis reaction between amine groups (-NH_2_) of gelatin and ABG components and N-hydroxysuccinimide (NHS)-ester of tetra-PEG-SG polymer, the hydrogel networks were formed within seconds, offering a multifunctional performance, including easy injection, favorable biocompatibility, biological and mechanical properties (compressive strength: 4.2 MPa; storage modulus: 10^4^ kPa; adhesive strength: 56 kPa), which could facilitate the stem cell viability, proliferation, migration and differentiation into osteocytes. In addition, the integration between the SGgel network and ABG moieties within a nano-scale level enabled the hybrid hydrogel to form adhesion to tissue, maintain the durable osteogenesis and accelerate bone regeneration. Therefore, a robust approach to the simultaneously satisfying tough adhesion onto the tissue defects and high efficiency for bone regeneration on a mouse skull was achieved, which may represent a promising strategy to design therapeutic scaffolds for tissue engineering in clinical applications.

## 1. Introduction

Bone tissue possesses unique self-repair and regeneration capacities for minor injuries, however, severe bone fractures and defects, as well as trauma and osteosarcoma, are still harmful diseases in orthopedics [1,2,3]. Although significant progress in surgical techniques has been made in clinic, delayed union or nonunion usually occurs in 10% of patients, resulting in disability and significant socioeconomic burdens [4,5,6,7]. Efforts are being made to improve the surgical treatment of autologous and allogeneic bone grafts for critical bone defects, however, these can cause clinical complications such as donor shortage, delayed operation time, limited donor volume, immune rejection of donor sites, and infection [8,9,10,11]. To tackle these shortcomings, the advent of the tissue engineering technique has provided creative ideas for promoting bone regeneration, on the basis of its unique advantages of exogenous progenitor cells and tailored release of biological agents, wherein the rapid development of biocompatible scaffolds with sufficient mechanical strength, matched degradation rate and promoted osteogenic activity has evolved as a primary topic in bone tissue engineering research [12,13,14,15,16,17,18].

Hydrogels comprising of 3D crosslinking polymer networks with multifariously physical structures and chemical properties have been widely explored as biological scaffolds to mimic the natural ECM environment when properly designed, thus facilitating the viability, growth, proliferation, and differentiation of the stem cells for tissue repair [19,20,21,22,23,24,25,26]. Nevertheless, most hydrogel scaffolds cannot simultaneously satisfy the multiple requirements of robust mechanics, cell compatibility and biological osteogenesis, thus hindering the clinical therapy of bone defects [27,28,29,30]. Moreover, an ideal scaffold should also possess a suitable porous architecture to offer sufficient biological immobilization space between the implanted scaffolds and regenerative tissues for adequate bone induction and angiogenesis.

Using high performance hydrogels as biological materials for bone tissue repair has led to great progress in enhancing the robustness of bone tissue in recent years. In general, the incorporation of bioactive components (e. g. POSS, BG, clay, HAp) is an effective method to improve the biological performance of bone constructs [31,32,33,34,35,36,37,38]. Wherein, bioactive glass (BG), mainly consisting of silicon, calcium, and phosphorus elements, has more capabilities on inducing the osteo-stimulation and bone mineralization for defect repair [39,40,41]. It was previously believed that, along with the implanted hydrogel scaffold degradation in vivo, the bioactive nanoparticles of hydroxyapatite or BG could be exposed-contacted into and subsequently bound onto the tissues during the degradation process in order to facilitate the angiogenesis, which was of vital importance to enhance bone regeneration [42,43]. Therefore, it is likely that the bioactive components may gradually degrade into the main biological components of the silicon, calcium and phosphorus elements, which could be absorbed in situ to promote bone mineralization. In particular, BG can be induced to produce hydroxyapatite layers by chemically binding to bone under physiological conditions, and analogously to the bone mineral stage, providing a favorable environment for the activity, growth, proliferation, and differentiation of osteoblast-related cells [44]. However, complex microenvironments in vivo can cause the explicit microphase separation between the inorganic phase and organic network, which endangers the long-term stability, durability, integrity, and osteogenic potentials of these nanocomposite materials, thus resulting in the disharmony between bone ingrowth and constructs degradation [45,46]. Therefore, the pursuit of advanced strategies for the achievement of long-term stability and maintenance of phase integration in vivo are urgently required in order to develop more intelligent scaffolds to guide bone regeneration.

**Figure 1:**
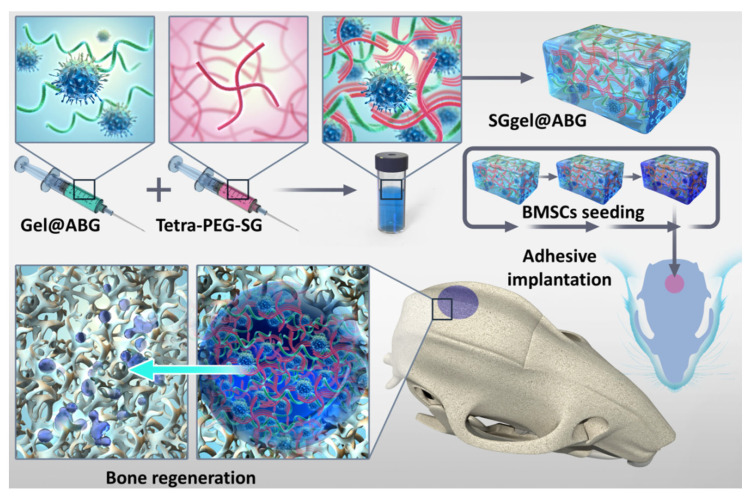
Schematic illustration of the fabrication procedures of SGgel@ABG composite hydrogel for calvaria bone defects repair.

In this work, in order to fabricate an advanced osteogenic scaffold that could simultaneously satisfy the multiple requirements of strong adhesion onto tissue defects, good compatibility, high mechanics and promote the sustainable osteogenesis effect, a class of highly integrated inorganic-inorganic hydrogel scaffold (SGgel@ABG) was developed via the rapid ammonolysis reaction between NHS-activated tetra-PEG-SG polymer and gelatin in the presence of an amine-terminated bioactive glass (ABG) particle in the one-pot strategy (Figure 1). On account of the powerful integration between the biological SGgel network and ABG moieties within a nano-scale level, the significant enhancement of the mechanics and osteogenic activity enabled SGgel@ABG composite hydrogel to form adhesion to the tissue and to maintain the durable osteogenesis and accelerate bone regeneration. This not only offered the surgeon the injectable and shapeless ability to fill in the gaps of different patterns, but also provided stable and sustained osteogenic activity that guided long-term bone growth in vivo, thereby indicating the potential therapeutic efficiency for the bone-related disease in clinic.

## 2. Results and Discussion

### 2.1. Preparation and Characterization of SGgel and SGgel@ABG Hydrogels

**Figure 2:**
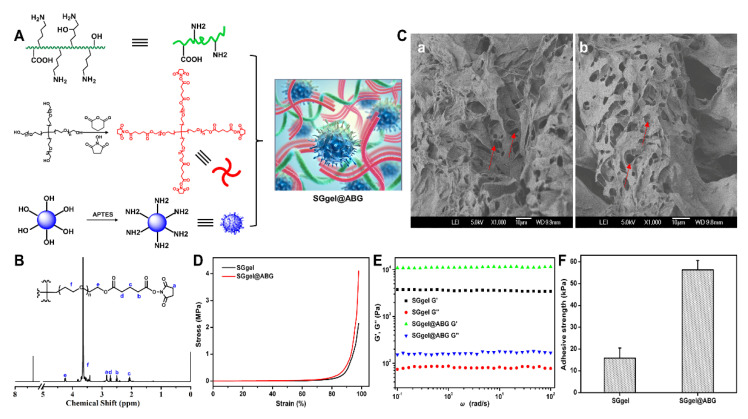
Structure and property characterizations. (**A**) Synthesis route of modified polymers and amine-functionalized ABG. (**B**) ^1^H NMR spectrum of the tetra-PEG-SG polymer. (**C**–**F**) SEM images, compressive, rheology and adhesive profiles of (**a**) SGgel and (**b**) SGgel@ABG hydrogels. Red arrows represent the similar inner pores.

The schematic illustration of the SGgel@ABG hybrid hydrogel was shown in Figure 2A. In order to avoid the misgivings of inhibition by anticoagulants and disease transfer, the tetra-PEG-SG was prepared using two steps, with high efficacy. All of the characteristic peaks in the ^1^H NMR spectrum could be found and belonged to its explicit molecular structure and the clear integration peak ratio (a/b/c/d = 2:1:1:1), as shown in Figure 2B, revealing the precise preparation of tetra-PEG-SG. On the basis of efficient ammonolysis reactions between the active ester groups and the amine in mild conditions, the SGgel hydrogel could be quickly obtained via simultaneously mixing the polymeric gelatin and tetra-PEG-SG solutions using a dual syringe. It was mentioned that the high adhesive strength of SGgel hydrogel with the biological tissue was also achieved by forming the chemical linkage among the amine-terminated proteins tissue and the tetra-PEG-SG polymers, which demonstrated its facile and accessible usage for the bone diseases with unique advantages, such as accessible injection behavior, short gelation time and powerful tissue adhesion. Although the residual active ester group of the SGgel hydrogel can provide adhesive strength on the amine-abundant tissues or other adherends, its cohesion strength was too weak to maintain the tough and durable adhesion to bone tissue. Therefore, a conventional hybridization method with BG moieties could furnish the composite hydrogel with tough cohesion onto the tissue and a strong osteointegration effect. However, the weak interactions between the inorganic phases and polymeric materials within the hybrid hydrogels could impair the mechanical toughness under exposure onto the external force. Therefore, a nanomodification strategy for improving the interface bonding strength of the inorganic-organic phase was proposed to construct polymeric nanocomposites via the amine functionalization by the coupling agent of APTES. In regard to the functionalized BG with abundant amine groups, the obtained ABG was simultaneously integrated into an organic network via the physical aggregation effect and chemical ammonolysis reaction with the ester-active tetra-PEG polymer. This spontaneously strengthened the adhesion and cohesion interactions and formed a highly integrated inorganic-organic structure of the SGgel@ABG hydrogel. In addition, due to the introduction of the inorganic ABG component, the swelling rate of the SGgel@ABG hydrogel declined in comparison to the traditional PEG-based hydrogels. In addition, the less humid environment in the defected bone also hindered the swelling behavior of the hydrogels. Therefore, the involved ABG moieties in the hydrogels were not only beneficial for epithelialization and collagen synthesis in chronic healing, but also retained its bioactivity in stimulating osteogenesis and angiogenesis at the target site.

The SEM images showed the morphologies of the hydrogel scaffolds, as can be seen in Figure 2C, in which the SGgel and SGgel@ABG hydrogels displayed similar architectures and inner pores (red arrow). These composite hydrogels had large pore diameters and uniform porosity, enabling the entry of host cells and the exchange of nutrients and metabolic wastes. Under this circumstance, the prepared SGgel@ABG hybrid hydrogel may enable the sustained ABG release, cell infiltration and differentiation, as well as the internal and external substance exchange of hydrogels. The compressive behavior was a key parameter in estimating the stability, mechanical stability, and practicability of the composite scaffolds. As shown in Figure 2D, compared to the SGgel, the SGgel@ABG hydrogel exhibited higher mechanical performances as a result of the introduction of rigid ABG, uniform distribution and modulus enhancement. Moreover, rheology was also applied to verify the rationalized network and optimized incorporation of bioactive ABG moieties on improving the mechanical properties of SGgel@ABG hydrogel (Figure 2E). The storage modulus G’ (10^4^ kPa), which is larger than G″, indicated an elastic state and rigid backbone, which is indicative of its potential values for the tissue regenerations.

Figure 2F showed that the SGgel@ABG hydrogel adhesive possessed a significant improvement in adhesive strength and tough cohesion compared to the SGgel hydrogel, which was attributed to the optimal involvement and uneven arrangement of ABG particles within the network, through both the physical absorption and chemical anchor. The rational design and ratio of the SGgel@ABG adhesive was relative to the cohesiveness and adhesiveness. The SGgel adhesives possessed weak mechanical strength due to its poor cohesiveness. It has been stated that, although the addition of ABG can significantly enhance the cohesiveness of the adhesive, it also depletes the NHS-ester groups for the adhesiveness in the SGgel. Therefore, over-dosed ABG could exhaust NHS-ester groups and decline the adhesiveness of the SGgel@ABG hydrogel adhesive. Taking both the cohesiveness and adhesiveness of hydrogel adhesive into consideration, it was concluded that the SGgel@ABG, containing 5% *w/v* of ABG, was an optimized formula with optimal structures and well-balanced properties. Therefore, with facile injection, tough adhesion and biological properties, this hydrogel adhesive may spark innovative clinical treatments to guide bone regeneration.

### 2.2. Cell Viability and Proliferation

**Figure 3:**
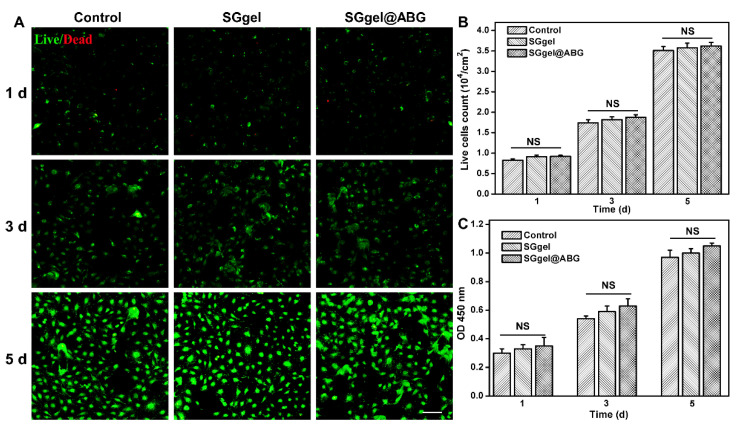
Cell cytotoxicity of SGgel and SGgel@ABG hybrid scaffolds in vitro. (**A**) Live/dead staining of BMSCs. Wherein, the green cells are the living BMSCs, and the red cells are the dead BMSCs. (**B**) Cell viability and (**C**) Cell proliferation of SGgel and SGgel@ABG hybrid scaffolds after the cultivation for the appointed time. NS, not significant.

Biomaterial compatibility is a prominent prerequisite for high-qualified cell carriers. The FDA-approved PEG and biocompatible gelatin are widely used in biomedical applications, such as in neural, articular cartilage tissue, bladder and bone repair. Therefore, cytotoxicity was first conducted to assess the safety of the SGgel and SGgel@ABG hydrogel scaffolds, by the BMSCs, using a live/dead assay. Figure 3A confirms the excellent cell biocompatibility with a clear observation of high BMSCs viability. Quantitatively, this ABG-conjoined SGgel could not only keep the favourable cell survival rate, but also significantly facilitated the cell growth after 5 days of in vitro culture, revealing its low toxicity on BMSCs. This is a preliminary indicator for the implantation of hydrogel scaffolds in the tissue engineering fields. The BMSCs proliferation experiment was further performed after the in vitro culture (1, 3 and 5 days) on each group. As expected, cell proliferation continued to increase in all of the groups, within the five days. As shown in Figure 3B, the density of BMSCs within the SGgel@ABG hydrogel scaffold was measured to be (0.83 ± 0.03) × 10^4^ cells/mL on day 1, (1.74 ± 0.08) × 10^4^ cells/mL on day 3 and (3.51 ± 0.1) × 10^4^ cells/mL on day 5, higher than those of the SGgel hydrogel and control group, indicating the important roles of ABG substrate on improving cell proliferation after prolonged incubation.

To explore whether this hybrid hydrogel could support cell activity, growth, and proliferation, we performed the proliferation experiment and measured the OD values at 450 nm after the in vitro culture on the hydrogel scaffolds using a CCK8 assay. After the co-culture of the SGgel@ABG hydrogel, the cell proliferation rate was slightly improved initially, and significantly increased at days 3 and 5 of in vitro incubation (Figure 3C), revealing the satisfactory cell growth and proliferation behavior of the ABG-conjoined SGgel hybrid scaffold.

### 2.3. In Vitro Osteogenic Differentiation

**Figure 4:**
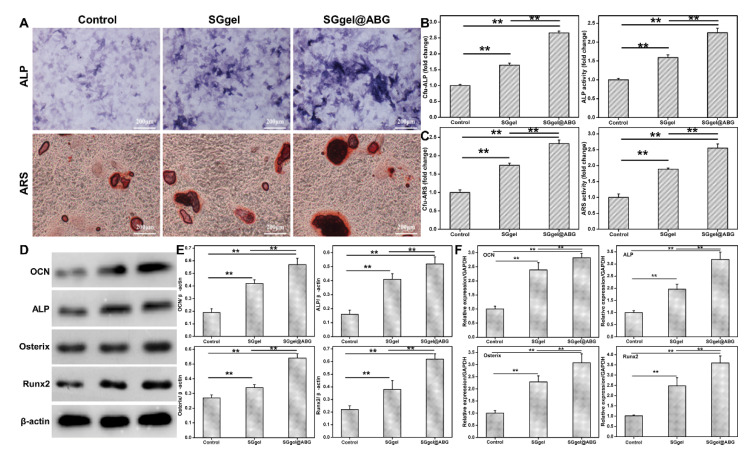
In vitro osteogenic differentiation of SGgel@ABG hydrogel. (**A**–**C**) ALP (14 d) and ARS staining (21 d) revealing the enhanced osteogenic differentiation of BMSCs. (**D**,**E**) Western blotting analysis and (**F**) qPCR quantification showing the highest osteogenic expression markers (OCN, ALP, Osterix and RUNX2) in the hydrogels. Statistically significant differences in comparison with untreated cells (control), SGgel hydrogel and SGgel@ABG hydrogel. ** *p* < 0.01.

In addition to the requirement good biocompatibility, ideal engineered scaffolds should also meet the needs of boosting progenitor cell’s osteogenic differentiation. ALP staining, ARS staining and qPCR were applied to assess the osteoinductivity of the SGgel@ABG hydrogel scaffold. As shown in Figure 4A–C, ALP staining and ARS staining revealed that the SGgel@ABG group possessed the greatest staining intensity and ratios of staining positive areas, compared to the other groups. These results indicated that the ALP activity at day 14 and the mineralized nodules at day 21 were conspicuously enhanced, demonstrating that the BMSCs cultured on the SGgel@ABG hydrogel could be activated and could enhance osteogenic differentiation. As an early osteogenic marker, the increasing ALP expressions within the hydrogel scaffold had a certain guiding significance for the osteogenic differentiation (Figure 4B). OCN, Osterix and RUNX2 were another three crucial markers of the BMSCs that play an important role in regulating bone metabolism, and their upregulated levels illustrated high-grade mineralization. The relative mRNA expressions of osteogenic marker genes (OCN, ALP, Osterix and RUNX2) were analyzed at various stages of osteogenesis. RT-qPCR was used to examine their osteoinduction abilities. Compared to the control group, ALP expression was significantly upregulated at each time point for the SGgel and SGgel@ABG groups, indicating their favorable components, architectures and mechanics for ossification effects. Intriguingly, it was shown that the expression of the osteogenesis-related gene markers of the SGgel@ABG group were more conspicuously increased than those in the SGgel group (Figure 4D), indicating the promotion of cell differentiation by favorable ABG moieties, as well as its uniform distribution, controlled release and improved mechanical properties. The highest upregulated expression after 7 d further implied that the ABG-laden SGgel hydrogel scaffold was capable of impelling BMSCs osteogenic differentiation in vitro. Collectively, these findings reinforced the statement that the SGgel@ABG hydrogel scaffold could effectively promote the osteogenic differentiation that is important for therapeutic ossification and bone regeneration in vivo.

### 2.4. Hybrid Hydrogel Scaffolds Regenerate Bone Formation In Vivo

**Figure 5:**
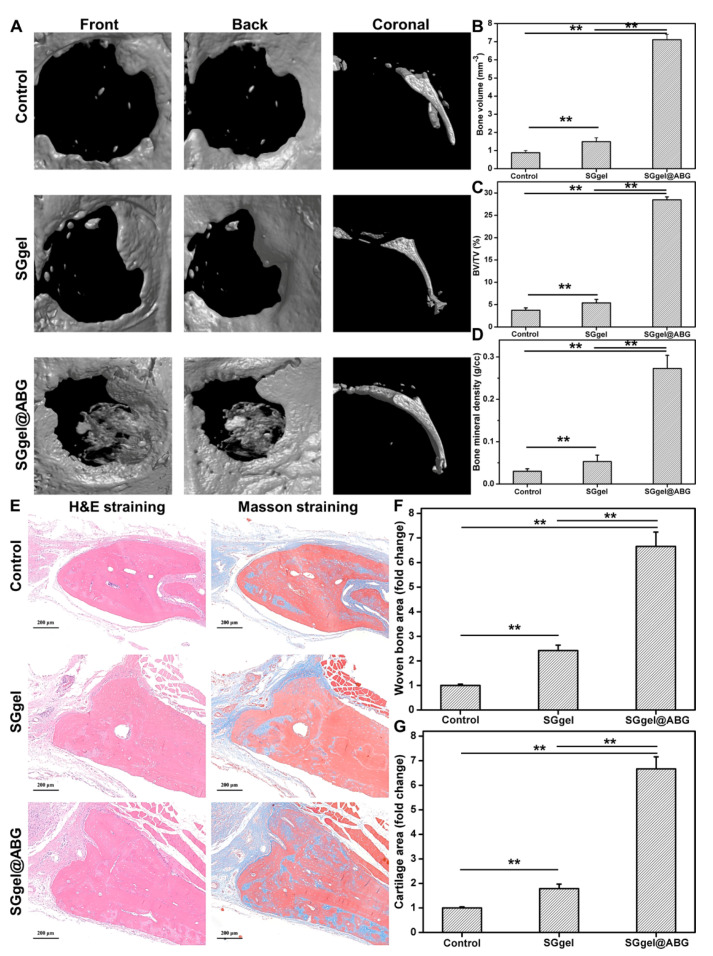
(**A**) 3D reconstruction of Micro-CT images of regenerated bone formation in rat cranium after the hydrogel implantation for 8 weeks with control group, SGgel group and SGgel@ABG group. (**B**–**D**) Quantitative analysis of BV, BV/TV and BMD of newly formed bone tissue. (**E**) H&E and Masson’s trichrome staining. (**F**,**G**) Woven bone and cartilage areas were analyzed in defect bone region. ** *p* < 0.01.

Inorganic BG is a series of bioactive, biodegradable and osteoconductive materials for bone tissue engineering, but its application in bone tissue engineering is limited due to its easy removal from the target site. In regard to the SGgel@ABG composite hydrogel with good mechanical, compatible and biological properties in vitro, we performed in vivo bone-defect experiments of this bioactive scaffold to demonstrate its strong bonding capacity, excellent cell ingrowth and regenerative bioactivity. A circular bone piece was fixed within freshly formed cranial defects and treated with the SGgel and SGgel@ABG hydrogels. The stability and bone regeneration of the circular bone plates were evaluated eight weeks after surgery. The micro-CT images exhibited the dislocation of the circular bone piece in the control group. This was the result of its lack of fracture fixation, sustainability and regenerative ability (Figure 5A). No similar phenotype was seen in the implanted hydrogel scaffolds, and the circular bone pieces were not fully healed in the SGgel group due to its insufficient nutrient and osteogenic supplies. By comparison, the SGgel@ABG group exhibited a good integration between the circular bone piece and its surrounding bone tissues. More importantly, its gap was conspicuously reduced at eight weeks, indicating the formation of vast new bones as a result of the bioactive ABG participant in the SGgel@ABG group. The microarchitectural parameters of the regenerated bone tissue within the cranial defect were confirmed by quantifying the bone volume within circular gap and the total volume within the circular gap (Figure 5B–D). The TV of the SGgel@ABG group was larger than that of the control and SGgel groups at eight weeks. The similar trend of BV/TV and BMD further indicated the achievement of the largest volume of new bone formation for the SGgel@ABG scaffold, which was consistent with the assumption of essential ABG introduction to satisfy the bioactive and mechanical support for new bone formation.

A histologic analysis experiment was carried out to differentiate the bone and soft tissue within the defect after eight weeks (Figure 5E–G). This was consistent with the micro-CT as soft tissue formation was observed but no new bone formation was observed in the control group, which implied the uneliminated the defect. In contrast, the SGgel@ABG group showed many fibrous connective tissues that had filled most of the defected areas. H&E staining showed the maximum bone regeneration in the defect area in the SGgel@ABG group, while less marginal bone formation was achieved in the SGgel group. The microstructure of newly formed bone in SGgel@ABG was relatively orderly and the distribution of osteocytes was more uniform, revealing the greatest abundant bone regeneration in the SGgel@ABG group. In addition, mature bone tissue was also detected in the targeted site to reveal its bone regeneration ability. Masson trichrome staining showed that the SGgel@ABG group had significantly more mature neo-bone than the SGgel group. The latter had more immature collagen fibers and osteoid blue-stained areas, and the former revealed the best quality of newly formed bone, indicating that ABG moieties could promote bone tissue growth. Thereafter, the cranial repair results proved its potential application as a suitable tissue-engineered scaffold.

The repair mechanism of the SGgel@ABG composite hydrogel may be attributed to the following factors: (1) the component of osteoid and bone tissues. Biocompatible natural gelatin was favored for the chondrogenesis to improve proteoglycan/collagen secretion, and polymeric tetra-PEG-SG provided a dense crosslinking junction and sufficient adhesion to the tissue surface to maintain the durable mechanical support and long-lasting stability; (2) the SGgel@ABG composite hydrogel could offer a moisture environment to ensure the surrounding cells’ survival, growth and proliferation, which played a role in the initial stage of bone regeneration; (3) in addition to the hydrogel degradation in vivo, ABG could be exposed-contacted into, and subsequently bound onto, the tissues to facilitate angiogenesis, which was of vital importance to enhance bone regeneration; (4) on account of the main biological components of silicon, calcium and phosphorus elements within the ABG, the gradual degradation and element release could be absorbed to promote bone mineralization. Consequently, in comparison to the previous attempts to fabricate tissue engineered hydrogel scaffolds for tissue regeneration, the current attempt presents a distinctive formula to offer a potential clinic alternative with good biocompatibility, facile injectability, usage flexibility and long-lasting osteogenic capacity.

## 3. Conclusions

In summary, we designed a novel SGgel@ABG hybrid hydrogel adhesive for local bone regeneration. The porous structure and favorable mechanics of the SGgel@ABG scaffold could enhance cell activity, growth, proliferation, and differentiation, while its strong adhesive strength and durable stability on bone tissue was essential for clinical bone repair. In addition, the amine-functionalized ABG moieties not only rationally provided the physical-chemical crosslinking junction on the improving the mechanical properties, but also significantly contributed to the bioactive osteogenic activity, which was found to effectively guide the osteogenesis, maintain the perdurability of bone formation, and accelerate the bone repair both in vitro and in vivo. This study demonstrated an adaptive strategy for producing bone scaffolds with a rational design and fabrication of advanced biomaterial hydrogels for clinical treatments.

## 4. Materials and Methods

### 4.1. Materials

Four-arm poly(ethylene glycol) (tetra-PEG-OH, M_w_ = 20 kDa, M_w_/M_n_ = 1.03, SINOPEG), glutaric anhydride (98%, Energy Chemical), N-hydroxysuccinimide (NHS, 98%, Energy Chemical), 3-aminopropyltriethoxysilane (APTES, J and K, 98%), gelatin (80–100 kDa, J and K), and bioactive glass (BG, 10–30 μm, biomedical grade) was purchased from Shanghai Aladdin Bio-chem Technology Co., Ltd. (Shanghai, China). All other chemical and biological compounds were purchased from Beijing Chemical Works (Beijing, China) and used without any further treatments and purification steps.

### 4.2. Measurements

The nuclear magnetic resonance spectrum was obtained on a Bruker DRX-400 spectrometer. Scanning electron microscopy (SEM) images were measured at an acceleration voltage of 5 kV, on a JSM-6700F microscope. The sample was sputter-coated with a thin layer of Pt for 120 s before the measurement. The rheological measurements were carried out on a Thermo Haake Rheometer, equipped with cone-parallel plate geometry (diameter: 35 mm), at a gap of 0.5. The sample was spread on a parallel plate, followed by sealing with the oil to prevent water evaporation during the experiment, which was conducted at 25 °C with a constant strain of 0.05%, in the frequency range of 100–0.1 rad s^−1^. The compressive testing was carried out by a universal Instron 3365 machine, at room temperature, and the hydrogels were cut into cylinders (diameter: 9 mm) with a velocity of 2 mm min^−1^. The adhesion testing was also performed using Instron 3365 at room temperature. One side of fresh squared porcine skins (diameter: 16 mm × 16 mm) was adhered to a Perspex cylinder using an α-cyanoacrylate glue, while the other side was adhered using the hydrogel adhesive at room temperature. When the attaching porcine skins were torn apart under the driven force, the shear strength was measured with a force sensor of 100 N.

### 4.3. Synthesis of Tetra-PEG-SG Polymer

First, tetra-PEG-OH (4 g, 0.2 mmol), DMAP (244 mg, 2 mmol) and glutaric anhydride (228 mg, 2 mmol) were dissolved in 50 mL of anhydrous CH_2_Cl_2_. After stirring for 24 h, the solution was washed with a hydrochloric acid aqueous solution, saturated sodium chloride aqueous solution and DI-water, several times over, and dried over anhydrous MgSO_4_. Next, the obtained product was further precipitated into diethyl ether for twice to obtain the tetra-arm PEG-glutaric acid polymer. Then, tetra-PEG-COOH (2 g, 0.1 mmol), NHS (460 mg, 4 mmol) and EDCI (768 mg, 4 mmol) were dissolved in 25 mL of anhydrous CH_2_Cl_2_. After stirring for 24 h, the solution was washed with a hydrochloric acid aqueous solution, saturated sodium chloride aqueous solution and DI-water, three times, and dried over anhydrous MgSO_4_ to yield the white solid of tetra-PEG-SG.

### 4.4. Synthesis of ABG

The modification method used to prepare ABG particle was carried out by adding 5 mL APTES into 100 mL of hexane, containing 0.3 g of BG. After stirring for 24 h at 60 °C, the amine-terminated ABG was facilely yielded after being washed with the ethanol and DI water several times.

### 4.5. Preparation of SGgel and SGgel@ABG Hydrogels

The SGgel adhesive was prepared by mixing the same volume of solution A, containing 20 wt% of gelatin, and solution B, containing 15% of tetra-PEG-SG polymer. For the SGgel@ABG, 5 wt% of ABG was incorporated into solution A with stable sonication, 10 min before mixing solution A and B.

### 4.6. In Vitro Cytotoxicity Assay

A CCK-8 assay was used to evaluate the cytotoxicity of the hydrogel scaffold. After suspending it into a fresh cell culture medium and seeding it into a 48-well plate, the BMSCs with a density of 1 × 10^4^ cells/100 µL were incubated for 24 h in a 5% CO_2_ humidified incubator. The hydrogel was treated in fresh cell medium for another 24 h in order to obtain the extracts. Then, the treated cell medium was used to replace the fresh cell medium to obtain further cell cultures. The cells that were cultured in the fresh medium formed the control group. The cell viability was calculated via the equation:

Cell viability (%) = [(A_sample_ − A_blank_)/(A_control_ − A_blank_)] × 100%

### 4.7. Live/Dead Staining Assay

The cell biocompatibilities were evaluated using a Live/Dead Viability Kit. The BMSCs on either a blank plate or hydrogels were stained with live/dead staining working solution (Calcein-AM/PI), and the stained cells were directly observed under the inverted optical microscope (Olympus, Japan). Calcein-AM generates a green fluorescence signal in living cells and PI only reaches the nuclei of dead cells to emit red fluorescence. The number of live cells was quantitatively analyzed using Image J software (Image J 1.52, Wayne Rasband, National Institute of Mental Health, Bethesda, Rockville, MD, USA).

### 4.8. Cell Proliferation Assay

Cell proliferation measurement was also carried out using a CCK-8 assay. Firstly, the BMSCs were incubated in a growth medium for 1 day. Next, the hydrogel samples were added into the medium and further incubated for another 1 day. After 5 days of cell incubation, 100 µL of fresh culture medium was used to replace the cell culture medium and added into 10 µL of CCK-8 for another 4 h. Then, the absorbance was measured at 450 nm and recorded, using a microplate reader to calculate the cell proliferation.

### 4.9. ALP Staining and ARS Staining

The BMSCs were seeded on the blank plate or the hydrogels pre-coated plate, with a density of 1 × 10^6^ cells/well in osteogenic induction medium. On differentiation days 14 and 21, the cells were washed several times, fixed with 4% paraformaldehyde for 15 min, and stained for 30 min using the ALP staining kit (day 14) and ARS solution (day 21). An inverted optical microscope was used to capture the images. The areas of ALP positive colony forming unit (Cfu-ALP) and ARS positive colony forming unit (Cfu-ARS) were calculated using Image J software. The stained cells were lysed with 10% hexadecylpyridinium chloride monohydrate for 15 min and the absorbance of the collected supernatant at 540 nm was measured using a spectrophotometer. The relative ALP and ARS activities were normalized to total protein content, which was determined using a BCA kit.

### 4.10. Quantitative Real-Time Polymerase Chain Reaction (qRT-PCR) Analysis

The total mRNA extraction was performed using TRIzol and was then subjected to reverse transcription by PrimeScript RT Master Mix. The qRT-PCR was conducted using the ABI 7500 Real-time PCR System (Atlantic Avenue, New York, CA, USA). The expression of the target genes was measured using TB Green Premix Ex Taq and the expression of β-actin was used as an endogenous control.

### 4.11. In Vivo Experiments

In order to study the osteogenesis effects of hydrogel scaffolds in vivo, the animal experiments were carried out on 8-week-old male Sprague−Dawley (SD) rats using the calvarial defect model. These rats were anesthetized by an intraperitoneal injection of pentobarbital (Nembutal 3.5 mg/100 g), and the sagittal incisions were made on the skin and the muscle to the cranial roof. After elevating the skin-muscle-periosteum flap, a 5 mm thick circular defect was created in the center of each parietal bone with a ring drill of a corresponding external diameter. Next, the implanted hydrogel samples were injected into the rat defects and the incisions were closed with the resorbable sutures. The rats returned to normal function after surgery. A total of 30 rats were randomly divided into the graft study groups: (1) negative control group (defect only), (2) SGgel/BMSCs group, and (3) SGgel@ABG/BMSCs group. All surgical interventions and postoperative animal care procedures were approved by the Animal Research Committee of Peking University (LA2020465).

### 4.12. Microcomputed Tomography (Micro-CT) Analysis

To assess the quality of the new bone formation, skull bones possessing a defect region were extracted 8 weeks after the implantation. Micro-CT was performed to scan the skull defects using the SkyScan 1076 System (Bruker, Kontich, Belgium), under a standard condition (40 kV X-ray voltage; 250 μA electric current; 18 μm per pixel). DataViewer was used to reconstruct 3D images of micro-CT. A diameter of 5 mm, covering the bone defect region in the reconstructed 3D image, was defined as the region of interest (ROI), and the bone morphometric parameters, including bone volume (BV), bone volume/tissue volume (BV/TV) and bone mineral density (BMD), were analyzed in ROI.

### 4.13. Histological Observations

The skull samples were fixed in 4% paraformaldehyde for 2 d and subsequently decalcified with a daily change of 15% tetrasodium EDTA for 28 d. From the center of the defect region in the coronal plane, a decalcified skull sample was divided into 8 mm thick slices. Next, the samples were dehydrated in an ethanol series before being embedded in paraffin. From the center of the defect region in the coronal plane, decalcified skull samples were sliced into 5 µm thick sections for the histological examination and morphological analysis with Hematoxylin-Eosin (H&E) staining and Masson’s trichrome staining.

### 4.14. Statistics Analysis

All the results were expressed in the form of mean and standard deviation and there were at least 3 independent experiments. For multi-group comparisons, the one-way ANOVA was assessed, followed by either the Student-Newman-Keuls or Dunnett’s test, wherever appropriate; *p* < 0.05 was considered statistically significant.

## Data Availability

Not applicable.
